# Supplementing Mannan Oligosaccharide Reduces the Passive Transfer of Immunoglobulin G and Improves Antioxidative Capacity, Immunity, and Intestinal Microbiota in Neonatal Goats

**DOI:** 10.3389/fmicb.2021.795081

**Published:** 2022-01-04

**Authors:** Chao Yang, Tianxi Zhang, Quanhua Tian, Yan Cheng, Kefyalew Gebeyew, Guowei Liu, Zhiliang Tan, Zhixiong He

**Affiliations:** ^1^CAS Key Laboratory for Agro-Ecological Processes in Subtropical Region, National Engineering Laboratory for Pollution Control and Waste Utilization in Livestock and Poultry Production, South-Central Experimental Station of Animal Nutrition and Feed Science in Ministry of Agriculture, Hunan Provincial Engineering Research Center for Healthy Livestock and Poultry Production, Institute of Subtropical Agriculture, The Chinese Academy of Sciences, Changsha, China; ^2^University of Chinese Academy of Sciences, Beijing, China; ^3^School of Environmental Ecology and Biological Engineering, Wuhan Institute of Technology, Wuhan, China; ^4^College of Animal Science and Technology, Southwest University, Chongqing, China

**Keywords:** neonatal goat, mannan oligosaccharides, passive immunity, IgG, intestinal microbiota

## Abstract

Successful establishment of passive immunity (PIT) and regulation of intestinal microbiota are crucial for ruminants to maintain body health and reduce the risk of disease during the neonatal period. Thus, the objective of this study was to investigate the effects of mannan oligosaccharide (MOS) supplementation on passive transfer of immunoglobulin G (IgG), serum inflammatory cytokines and antioxidant levels as well as bacteria composition in the ileal digesta. A total of 14 healthy neonatal Ganxi black goats with similar birth weight (BW: 2.35 ± 0.55 kg) were selected and allocated into two groups, only fed colostrum and milk replacer (CON, *n* = 7) and supplemented MOS (0.06% of birth BW) in the colostrum and milk replacer (MOS, *n* = 7). The results indicated that MOS supplementation significantly reduced (*p* < 0.05) serum IgG level at 3 and 6 h after colostrum feeding. Serum GLP-1 level of goats in the MOS group was significantly lower (*p* = 0.001) than that in the CON group. Goats in the MOS group had higher serum CAT and lower MDA level than those in the CON group (*p* < 0.05). Serum anti-inflammatory cytokine level of interleukin 4 (IL-4) was increased (*p* < 0.05), while pro-inflammatory cytokine IL-6 level was reduced (*p* < 0.05) in the MOS group when compared with the CON group. In addition, MOS supplementation remarkably increased (*p* < 0.05) the level of secretory IgA (sIgA) in the ileal digesta. Principal coordinate analysis of 16S rRNA sequence based on Brinary jaccard, Bray curtis, and weighted UniFrac distance of ileal microbiota showed a distinct microbial differentiation between the CON and MOS groups (*p* < 0.05). The relative abundance of *Firmicutes* in the MOS group was higher than that in the CON group, while the abundance of *Verrucomicrobia* was lower in the MOS group than that in the CON group at the phylum level (*p* < 0.05). The relative abundance of *Proteobacteria* tended to decrease (*p* = 0.078) in the MOS group at the phylum level. The results of LEfSe analysis showed that MOS group was characterized by a higher relative abundance of *Lactobacillus*, while the CON group was represented by a higher relative abundance of *Akkermansia* and *Ruminiclostridium_5*. Our findings demonstrated that MOS supplementation during the neonatal period increases antioxidant capacity and reduces the inflammatory response, and promotes IgA secretion and *Lactobacillus* colonization in the ileum. Thus, MOS induced positive effects are more pronounced in neonatal goats that might be an effective approach to maintain intestinal health and improve the surviving rate of neonatal ruminants.

## Introduction

The morbidity and mortality of newborn ruminants is the main factor that largely restricts the development of animal husbandry and farm profitability (Dwyer et al., 2016). In the last decades, the mortality is up to 50% in sheep and goats during the pre-weaning period (Thiruvenkadan and Karunanithi, 2007), and the mortality in kid goat ranges from 11.5 to 37% (Thiruvenkadan and Karunanithi, 2007; Singh et al., 2011). Neonatal animals are susceptible to infectious diseases, including respiratory disorders and gastrointestinal diseases, because their imperfect immune system exhibits a limited capacity to resist the invasion of pathogenic agents (Windeyer et al., 2014; Cheng et al., 2021). As one of the common digestive disorders, diarrhea causes high mortality rates ranging from 29 to 58% of neonatal calves, which are mainly infected by pathogens including *Escherichia coli*, *Salmonella*, and *Cryptosporidium* (Hunter and Thompson, 2005; Azizzadeh et al., 2012; Windeyer et al., 2014). Thus, it is essential to formulate an effective feeding regime to decrease the morbidity and alleviate the mortality rates of goat kids during the neonatal period.

Colostrum is rich in immunoglobulin, immune-stimulating peptides, and antimicrobial agents (Micha et al., 2020). It is a crucial vehicle for neonatal ruminants to establish passive immunity (PIT) due to their special synepitheliochorial placenta structure that does not empower the transfer of aforementioned immune factors from the dam to the fetus (Borghesi et al., 2014). The functions of colostrum in establishing PIT, intestinal bacteria colonization and prevention of diarrhea are mainly affected by the quality of colostrum and feeding time after birth (Morrill et al., 2012; Fischer et al., 2018). Furthermore, colostrum is very likely to be contaminated by collecting staff and instruments, which may increase the risk of intestinal diseases for calves (Godden et al., 2012). Heat treatment (60°C, 60 min) is a recognized approach to decrease pathogenic bacterial count and has little effect on immunoglobulin G (IgG) concentration (Donahue et al., 2012). Feeding heat-treated colostrum during the first 12 h after birth can enhance the colonization of *Bifidobacterium* and reduce the prevalence of *E. coli* in the small intestine of the calve (Malmuthuge et al., 2015). These results indicate the role of heat-treated colostrum in PIT transfer and beneficial bacteria colonization of ruminants, which can be used to maintain intestinal health during the neonatal period.

Mannan-oligosaccharide (MOS) as a functional oligosaccharide is always widely used in the diets of pigs (Duan et al., 2016), hens (Bozkurt et al., 2016), and rabbits (Abdel-Hamid and Farahat, 2016) to improve growth performance and immunity. Supplementing MOS in the basal diets usually improves fiber digestion, nitrogen deposition, and antioxidant capacity in adult sheep (Zheng et al., 2018). MOS also enhances the immunity of cows infected by rotavirus and promotes the transfer of antibodies against rotavirus to their offspring (Franklin et al., 2005). Furthermore, MOS supplementation increases average daily gain (ADG) and inhibits the colonization of pathogenic and nonpathogenic *E. coli* in the intestine of young calves (Lucey et al., 2021). However, the information on the effects of MOS supplementation on IgG absorption, intestinal microbiota, and immunity in neonatal ruminants is limited.

In the current goat feeding system, the phenomenon that neonatal goats fail to intake adequate colostrum happens commonly due to the lower colostrum production of their dams. In addition, goat colostrum may contaminate by a severe infectious virus that induces Caprine Arthritis–Encephalitis (CAE) and increases the morbidity and mortality (Blacklaws et al., 2004). To ensure successful PIT transfer and minimize the risk of CAE for neonatal goats, bovine colostrum is widely used to instead of goat colostrum (Nordi et al., 2012). In the current study, we used heat treated bovine colostrum as active immune factors to feed the neonatal goats. The objective of this study was to investigate the effects of MOS supplementation on serum biochemistry, IgG absorption, antioxidant ability, immunity, and the colonization of ileal bacteria in neonatal goats.

## Materials and Methods

### Colostrum Collection, Process, and Chemical Composition Analysis

Due to the low yield and the difficulty in collecting colostrum from Ganxi black goats, we used dairy cow colostrum in this study, and the colostrum was collected from six multiparous dairy cows during 12 h after parturition in the dairy farm of the Institute of Hunan Animal and Veterinary Science (Changsha, China). Before colostrum collecting, surface skin of breast was scrubbed with 1% povidone-iodine (LIRCON, Shandong, China) and milking staff wore medical mask and sterile gloves to prevent colostrum from contamination. Colostrum from each cow was collected into a 5 L sterile plastic bag. All colostrum was fully mixed when the collection was finished. Subsequently, the mixed colostrum was pasteurized using a commercial pasteurizer of 30 L (ZUOLANBO, Shandong, China) at 60°C for 60 min followed by rapid cooling (Malmuthuge et al., 2015). After cooling, heat-treated colostrum was aliquoted into 50 ml sterile centrifuge tubes and stored at −20°C for feeding trial. In addition, 50 ml heat-treated colostrum was prepared to determine colostrum composition (total solids, protein, fat, and lactose) based on Milk Analyzers (FOSS electric, Hilleroed, Denmark). Approximate 5 ml heat-treated colostrum was centrifuged at 12,000 rpm for 15 min at 4°C to obtain supernatant to detect IgG, IgA, and IgM concentration using commercial bovine specific ELISA kits (CUSABIO).^1^ The heat-treated colostrum contained 32.00% total solids, 22.21% protein, 6.81% fat, 3.11% lactose, 28.61 mg/ml IgG, 3.73 mg/ml IgA, and 2.93 mg/ml IgM.

### Experimental Design and Management

The experimental procedures of this study were performed in accordance with the guidance of the Animal Ethics Committee of Institute of Subtropical Agriculture, Chinese Academy of Sciences. All neonatal goats used in this study were brought from a commercial farm (Jiangxi Mulei Agriculture and Forestry Development Co. Ltd., Jiangxi, China) and the animal trial was also conducted in this farm from November 2019 to July 2020. A total of 14 healthy neonatal Ganxi black goats with similar birth weight (BW: 2.35 ± 0.55 kg) were selected and used in this study. Neonatal goats that were naturally delivered were immediately separated from their dams after birth to avoid any physical contact. All neonatal goats were artificially removed afterbirth and their bodies were dried using sterile towels. Thereafter, umbilical cords of neonatal goats were sterilized by 7% povidone-iodine and BW was recorded before moving each animal to an individual pen (80 cm × 160 cm × 100 cm) equipped with a heat lamp and bedded with rice straw. The house and pen were cleaned and sterilized thoroughly before the animal trial. Neonatal goats were randomly allocated according to their BW into two groups for 7 days. In addition, none of the animals included in this study were received any vaccination or therapeutic medicine during the overall experimental period.

**Figure 1:**
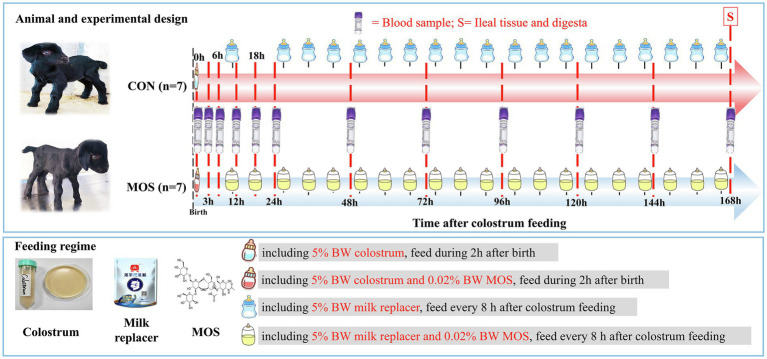
Experimental design, feeding regime, and sample collection.

The experimental design and feeding regime are illustrated in Figure 1. In details, neonatal goats were bottle-fed colostrum with a volume of 5% of BW at 2 h, and milk powder was diluted with water (at 42°C) in a ratio of 1:5 that was subsequently provided to goats with a volume of 5% BW every 8 h until the end of animal trial. The milk powder (contained 6% moisture, 23% crude protein, 12% crude fat, 3% crude fiber, 10% ash, 1.5% calcium, and 1.2% phosphorus) was purchased from Beijing Precision Animal Nutrition Research Center (Beijing, China).^2^ All neonatal goats were given *ad libitum* access to water. Otherwise, neonatal goats in the control group (CON, *n* = 7) only fed colostrum and milk replacer; however, those assigned to the treatment group (MOS, *n* = 7) were supplemented 0.06% of BW MOS (purity: 99%, FENGTAI, Shandong, China) in the colostrum and milk replacer per day throughout the experiment.

### Sample Collection

The blood sample was collected through the jugular vein using a heparinized tube (SANLI Medical Technology Development Co., Ltd., Changsha, China) at 0, 3, 6, 12, 18, 24, 48, 72, 96, 120, 144, and 168 h after colostrum feeding. After that, all blood samples were kept on the ice and taken to the laboratory for serum separation through centrifuging at 3,000 × *g* at 4°C for 20 min. The supernatants were collected and aliquoted into four 1.5-ml microcentrifuge tubes and stored at −20°C for determination of immunoglobulin and cytokine levels.

The neonatal goats were slaughtered after 168 h of colostrum feeding with an intravenous injection of sodium pentobarbital at 50 mg/kg BW for anesthetization. Subsequently, exsanguination was conducted until goats reached a surgical level of anesthesia. Immediately after opening the abdominal cavity, the proximal duodenum, and distal rectum were first ligated to avoid loss of digesta and then ligation was cautiously performed between segments to prevent the transfer of contents. In details, the ligated positions for ileum segments were from the ileocecal junction to 50 cm proximal to the ileocecal junction. Thereafter, ileal digesta was collected using 50 ml germfree centrifuge tubes (Corning®, NY, United States) and snap-frozen in liquid nitrogen and then stored at −80°C in the laboratory for further analysis. The tissues of aforementioned intestinal segments were washed in 100 ml sterile culture dishes slightly (Corning®, NY, United States) with ice-cold DNase/RNase-free ddH_2_O (R1600, Solarbio LIFE SCIENCES, Beijing, China) until clean (3–4 times). The tissues were cut into small pieces, then a part of tissues was transferred into 10% neutral buffered formalin for morphology observation, and other tissues were placed in a sterile sample bag (B01064, Whirl-Pak®, WI, United States), which immediately snap-frozen in liquid nitrogen and stored at −80°C for RNA extraction.

### Analysis of Biochemical Parameters and Hormone in Serum

The levels of glucose (GLU), triglyceride (TG), cholesterol (CHOL), low density lipoprotein (LDL), high density lipoprotein (HDL), blood urea nitrogen (BUN), and total protein (TP) in collected serum samples were measured using an automatic biochemical analyzer (Cobas c311, Roche, Basel, Switzerland). Serum hormone levels of growth hormone (GH), insulin (INS), insulin-like growth factor 1 (IGF-1), and glucagon-like peptide 1 (GLP-1) were determined using ELISA kits (CUSABIO; see Footnote 1) on microplate reader according to manufacturer’s protocols.

### Measurement of Immune and Antioxidative Indices in Serum

The concentration of IgG in serum was determined by using a commercial goat specific ELISA kit (CUSABIO, Wuhan, China) and the concentrations of IgA and IgM were analyzed using commercial sandwich ELISA technique kits (Jiangsu Meimian industrial Co., Ltd., Yancheng, China). The serum levels of interleukin-4 (IL-4), IL-6, IL-10, IL-12, interferon-β (INF-β), and tumor necrosis factor-α (TNF-α) were detected using the goat-specific ELISA kits according to manufacturer’s specifications (Jiangsu Meimian industrial Co., Ltd., Yancheng, China). The detection of serum antioxidant capacity, including superoxide dismutase (SOD), glutathion peroxidase (GSH-Px), catalase (CAT), total anti-oxidation capability (T-AOC), and malondialdehyde (MDA) levels were performed using the commercial kits according to the manufacturer’s protocols (Nanjing Jiancheng Bioengineering Institute, Nanjing, China).

### Ileal Morphology Analysis

After being fixed in the buffered formalin for 48 h, the ileal tissue sample was washed, dehydrated in gradient ethanol, and finally embedding in the paraffin wax. Five sections for each sample were sliced, installed on glass slides and stained with eosin and hematoxylin. Three random straightest villi and their accompanying crypts of each slide were selected to measure the villus height and crypt depth using a fluorescence microscope (Olympus, Tokyo, Japan) with a calibrated 10-fold eyepiece graticule. The V/C value (ration of villus height to crypt depth) was calculated.

### Secretory IgA Determination in the Ileal Digesta

Approximately 500 mg digesta samples of ileum were weighed in 2-ml microtubes and vacuum freeze-dried for 3 h at 1,200 rpm in a vacuum freeze drier (Lyovapor™ L-300, BUCHI, Switzerland). Subsequently, 80 mg of each dried sample were weighed in a new 2-ml microtube and added 2 ml of PBS (containing 0.05% Tween and protease inhibitor cocktail) to suspend it. Digesta samples were then thoroughly artificially shaken and mechanically homogenized on a vortex mixer until full resuspension. The supernatants were collected in a new tube after centrifugation at 3,000 × *g* for 10 min, and the concentration of secretory IgA (sIgA) of digesta samples was detected using a commercial goat specific ELISA kit according to manufacturer’s direction (Jiangsu Meimian industrial Co., Ltd., Yancheng, China).

### 16s rRNA Gene Sequencing and Bioinformatics Analysis

Microbial DNA extracted from 14 ileal digesta samples were performed using a commercial kit (DP328, TIANGEN BIOTECH Co., Ltd., Beijing, China) following the manufacturer’s instructions. The integrity and concentration of the extracted DNA were evaluated by 2.0% agarose gel electrophoresis and NanoDrop 2000 (Thermo Scientific, MA, United States), respectively. The primers 343F (5′-TACGGRAGGCAGCAG-3′) and 798R (5′-AGGGTATCTAATCCT-3′) of the V3–V4 variable region of the bacterial 16S ribosomal RNA gene was used for amplification on a ProFlex PCR system (ThermoFisher Scientific Inc., MA, United States). The amplified PCR products were verified by 2% agarose gels and then purified using the AxyPrep DNA Gel Extraction Kits (Axygen Biosciences, CA, United States) according to the manufacturer’s protocols. Purified PCR products were quantified by Qubit®3.0 (Life Invitrogen), and that for each sample were mixed equally to construct Illumina pair-end libraries following Illumina’s genomic DNA library preparation procedure. The results showed that the quality of the DNA library for two samples (one from the CON group and one from the MOS group) was ineligible. Subsequently, eligible amplicon libraries were sequenced on an Illumina MiSeq platform (PE250, Illumina, CA, United States) by Shanghai BIOZERON Co., Ltd (Shanghai, China). The raw reads were deposited into the NCBI Sequence Read Archive (SRA) database (Accession Number: PRJNA761090).

The quality control of raw data in FASTQ files was performed in QIIME data analysis package (version 1.9.1; Caporaso et al., 2010). In details, raw paired-end reads with 10 bp of minimal overlapping, the length less than 200 bp, average quality score less than 20, and 20% of maximum mismatch rate were excluded and the ambiguous nucleotides and chimeras were also discarded. Clean reads were then clustered into operational taxonomic units (OTUs) at 97% similarity cut-off using USEARCH (version 10).^3^ The representative read of each OTUs was annotated and aligned to the SILVA 16s rRNA database (version 138.1) using the RDP classifier algorithm with a confidence threshold of 80% (Quast et al., 2012). The rarefaction analysis based on Mothur (version 1.44.1) was conducted to reveal the diversity indices including the Chao1, ACE, Simpson, and Shannon indices. The beta diversity analysis was performed using principal coordinates analysis (PCoA) based on Binary jaccard, Bray curtis, and weighted UniFrac distance and the analysis of similarity (ANOSIM) was used to assess the differences among samples. The linear discriminant analysis (LDA) effect size (LEfSe) tool was applied to understand microbial communities through identifying different taxa between the CON and MOS groups using online packages (LC-Bio Technology Co., Ltd., Hangzhou, China) and using the threshold value (*p* < 0.05 and LDA > 2). The inferred metagenomic metabolic function of ileal microbiota was analyzed by PICRUSt2 based on normalized OTU abundance and the Kyoto Encyclopedia of Genes and Genomes (KEGG) pathways on level 3 were used to further analysis (Langille et al., 2013). The STAMP software (version 2.1.3) was employed to identify the different pathways between the CON and MOS groups using Welch’s *t*-test, and *p* value was adjusted by the Benjamini and Hochberg method (Parks et al., 2014).

### RNA Extraction and Quantitative RT-PCR

Total RNA extraction from ileal tissue sample was performed using SteadyPure Universal RNA Extraction Kit (AG21017, Accurate Biology, Changsha, China) according to the manufacturer’s instructions and then genomic DNA was removed using DNase I (Accurate Biology, Changsha, China). The quality and concentrations of isolated total RNA were detected using a NanoDrop 2000 (Thermo Scientific, MA, United States), and the integrity was verified by 1.0% agarose-formaldehyde gel electrophoresis. Thereafter, cDNA synthesis was performed using a commercial *Evo M-MLV* Reverse Transcription Kit [AG11705, Accurate Biology (Hunan) Co., Ltd., Changsha, China] and stored at −20°C for subsequent quantitative RT-PCR.

The expression of genes related to tight junction and immunity were determined using SYBR® Green Premix *Pro Taq* HS qPCR Kit [AG11701, Accurate Biology (Hunan) Co., Ltd., Changsha, China] based on a fluorescence LightCycler 480 II platform (Roche, Basel, Switzerland) according to the manufacture’s specification. The reaction program was described by Yang et al. (2021). The expression of each candidate genes was calculated based on quantification cycle normalized by housekeeping genes (*GAPDH* and *β-actin*) using the 2^-ΔΔCt^ method (Schmittgen and Livak, 2008). The primer information for candidate genes and housekeeping genes was deposited in Supplementary Table S1.

### Statistical Analysis

The results of biochemical, immune, and antioxidative indices in serum, morphologic parameters, and gene expression in the ileum, sIgA concentration and alpha-diversity index in the ileal digesta were analyzed using the independent-sample *t*-test in SPSS software (SPSS version 25.0, SPSS, Inc.). In addition, the data including the relative abundance of bacteria at the phylum and genus levels in the ileal digesta samples disobeyed normal distribution, Wilcoxon rank-sum test was applied to identify differential taxa. Data were presented as means with SEM. Value of *p* < 0.05 was regarded as statistically significant, and 0.05 ≤ *p* < 0.10 was regarded as a statistical tendency. The correlation analysis between the phenotypic values (including cytokines and antioxidative indices in serum and sIgA concentration in ileal digesta) and main bacteria (average relative abundance more than 0.1% in at least one group) was conducted using Spearman rank correlation coefficient in R package and heatmap was generated on OmicStudio (LC-Bio Technology Co., Ltd., Hangzhou, China). The significant correlation was identified by the threshold value *p* < 0.05 and |*r*| > 0.6.

## Results

### Serum Biochemical Parameters and Hormone Levels

As shown in Table 1, no difference (*p* > 0.10) was observed between the CON and the MOS group for serum GLU, TG, CHOL, LDL, HDL, and BUN. Neonatal goats in the MOS group had lower (*p* = 0.001) serum GLP-1 level than those in the CON group; however, there was no significant difference (*p* > 0.10) between the CON and the MOS group for serum GH, INS, and IGF-1 levels.

**Table 1:** Serum biochemical parameters and hormone levels of neonatal goats with MOS supplementation.

Item	Treatment		SEM	*p*-value
	CON	MOS		
GLU (mmol/L)	6.357	6.543	0.313	0.780
TG (mmol/L)	0.741	0.761	0.104	0.928
CHOL (mmol/L)	2.697	2.704	0.076	0.965
LDL (mmol/L)	0.873	0.799	0.048	0.458
HDL (mmol/L)	2.127	2.237	0.052	0.306
BUN (mmol/L)	4.643	4.371	0.334	0.701
GH (ng/ml)	12.400	15.150	1.165	0.253
INS (μlU/ml)	11.149	10.924	0.535	0.843
IGF-1 (ng/ml)	88.025	86.718	0.909	0.494
GLP-1 (ng/ml)	4.829	3.772	0.188	0.001

*Values are expressed as means ± SEM, n = 7. p < 0.05 was regarded as statistically significant, and 0.05 < p < 0.10 was regarded as a statistical tendency*.

### Serum Immunoglobulins, Antioxidant Status, and Inflammatory Cytokines

**Figure 2:**
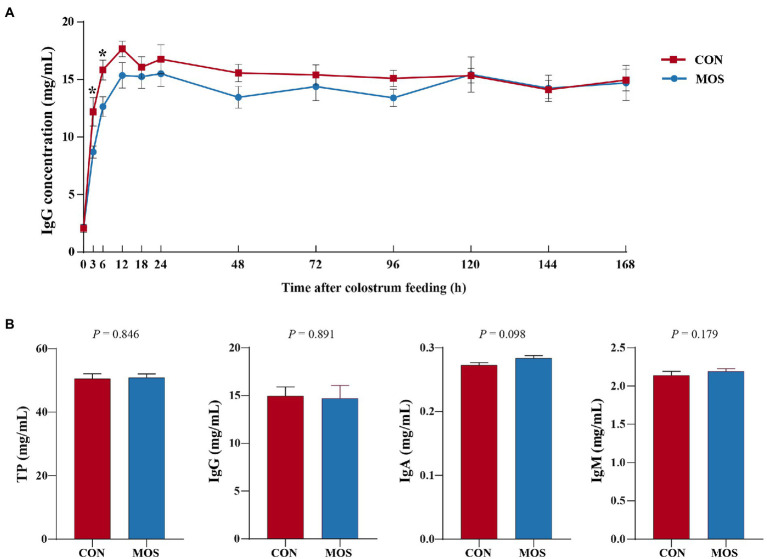
Serum immunoglobulin level of neonatal goats in the CON and mannan oligosaccharide (MOS) groups. **(A)** Serum immunoglobulin G (IgG) level at different sample collecting time after colostrum feeding. *p < 0.05. **(B)** Serum content of total protein (TP), IgG, IgA, and IgM at the end of experiment.

To monitor the passive IgG transfer of neonatal goats, we collected the serum samples at 0, 3, 6, 12, 18, 24, 48, 72, 96, 120, 144, and 168 h after colostrum feeding. Neonatal goats fed with MOS had significantly lower IgG concentration at 3 (*p* = 0.023) and 6 h (*p* = 0.022) when compared with the CON group (Figure 2A). Furthermore, the serum levels of TP, IgG, and IgM were similar (*p* > 0.10) in the CON and MOS groups, while IgA concentration tended to be higher (*p* = 0.098) in the MOS group than that in the CON group (Figure 2B). Neonatal goats in the MOS group had higher (*p* = 0.001) enzyme activity of CAT than those in the CON group, and the serum level of MDA was higher (*p* < 0.001) in the CON group than in the MOS group (Table 2). As presented in Table 3, the serum cytokine concentrations of TNF-α, IL-12, and IL-10 had no difference (*p* > 0.10) between the CON and the MOS groups. Goats in the MOS group had lower (*p* = 0.002) level of pro-inflammatory IL-6 and remarkably higher (*p* = 0.008) anti-inflammatory IL-4 level when compared with the CON group. In addition, supplementation of MOS tended to decrease (*p* = 0.074) pro-inflammatory IFN-β concentration in the MOS group than in the CON group.

**Table 2:** Serum antioxidative indices of neonatal goats with mannan oligosaccharide supplementation.

Item	Treatment		SEM	*p*-value
	CON	MOS		
GSH-Px (U/ml)	104.271	105.692	8.325	0.936
SOD (U/ml)	27.048	29.450	1.043	0.266
CAT (U/ml)	0.869	4.754	0.633	0.001
T-AOC (U/ml)	0.892	0.898	0.062	0.964
MDA (mmol/L)	2.139	1.149	0.156	<0.001

*Values are expressed as means ± SEM, n = 7. p < 0.05 was regarded as statistically significant, and 0.05 < p < 0.10 was regarded as a statistical tendency*.

**Table 3:** Serum cytokine levels of neonatal goats with mannan oligosaccharide supplementation.

Item	Treatment		SEM	*p*-value
	CON	MOS		
IFN-β (pg/ml)	419.488	376.281	11.332	0.074
TNF-α (pg/ml)	179.543	189.282	2.998	0.106
IL-6 (pg/ml)	145.703	134.419	2.091	0.002
IL-12 (pg/ml)	913.851	841.244	24.715	0.148
IL-4 (pg/ml)	44.392	48.829	0.907	0.008
IL-10 (pg/ml)	45.547	46.025	0.766	0.769

*Values are expressed as means ± SEM, n = 7. p < 0.05 was regarded as statistically significant, and 0.05 < p < 0.10 was regarded as a statistical tendency*.

### Ileal Morphology and IgA Secretion in the Ileal Digesta

Mannan oligosaccharide supplementation had no effect (*p* > 0.10) on villus height, crypt depth, and V/C in the ileum of goats (Table 4; Supplementary Figure S1); however, the sIgA concentration of the ileal digesta was significantly elevated (*p* = 0.043) in the MOS group than in the CON group (Table 4).

**Table 4:** Gut morphology and sIgA level in the ileum of neonatal goats with MOS supplementation.

Item	Treatment		SEM	*p*-value
	CON	MOS		
Villus length (μm)	445.90	412.90	20.588	0.445
Crypt depth (μm)	122.62	141.31	7.313	0.214
V/C	3.66	3.02	0.215	0.148
sIgA (mg/g of digesta)	0.043	0.057	0.004	0.043

*Values are expressed as means ± SEM, n = 7. p < 0.05 was regarded as statistically significant, and 0.05 < p < 0.10 was regarded as a statistical tendency*.

### Quantitative RT-PCR for Tight Junction and Immune Related Genes in the Ileal Tissue

**Figure 3:**
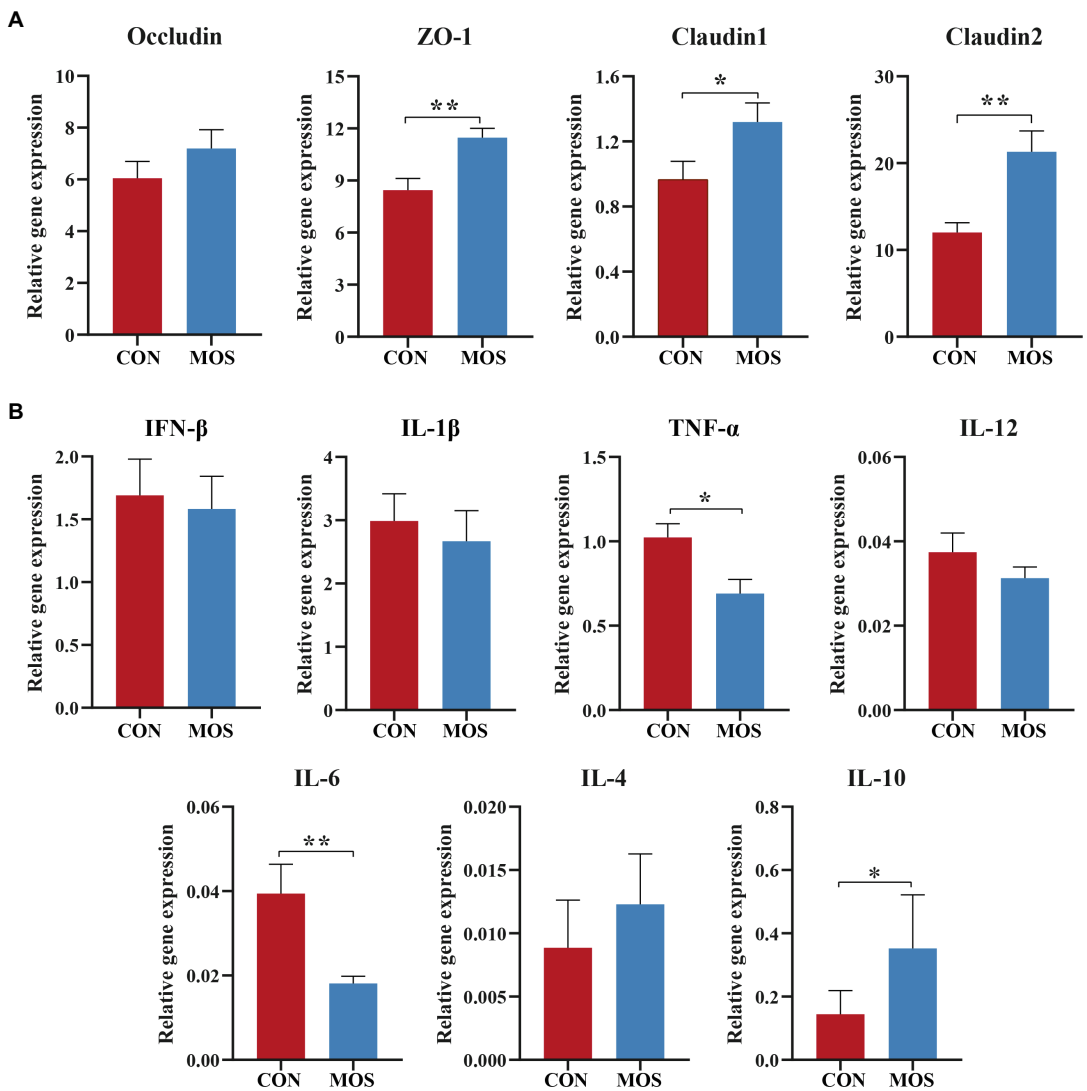
The relative expression of genes involved in **(A)** tight junction and **(B)** inflammatory response. *p < 0.05, **p < 0.01.

The mRNA expression of *Occludin* was not affected (*p* > 0.10) between the CON and MOS groups. However, increases in mRNA expression of *ZO-1* (*p* = 0.004), *Claudin1* (*p* = 0.049), and *Claudin2* (*p* = 0.008) were observed when MOS supplementation (Figure 3A). The relative expression of pro-inflammatory genes *TNF-α* (*p* = 0.015) and *IL-6* (*p* = 0.022) was higher in the CON group than those in the MOS group, while the mRNA abundance of anti-inflammatory *IL-10* was higher (*p* = 0.017) in the MOS group than that in the CON group (Figure 3B).

### Diversity and Taxonomic Composition of Microbiota in the Ileal Digesta

**Figure 4:**
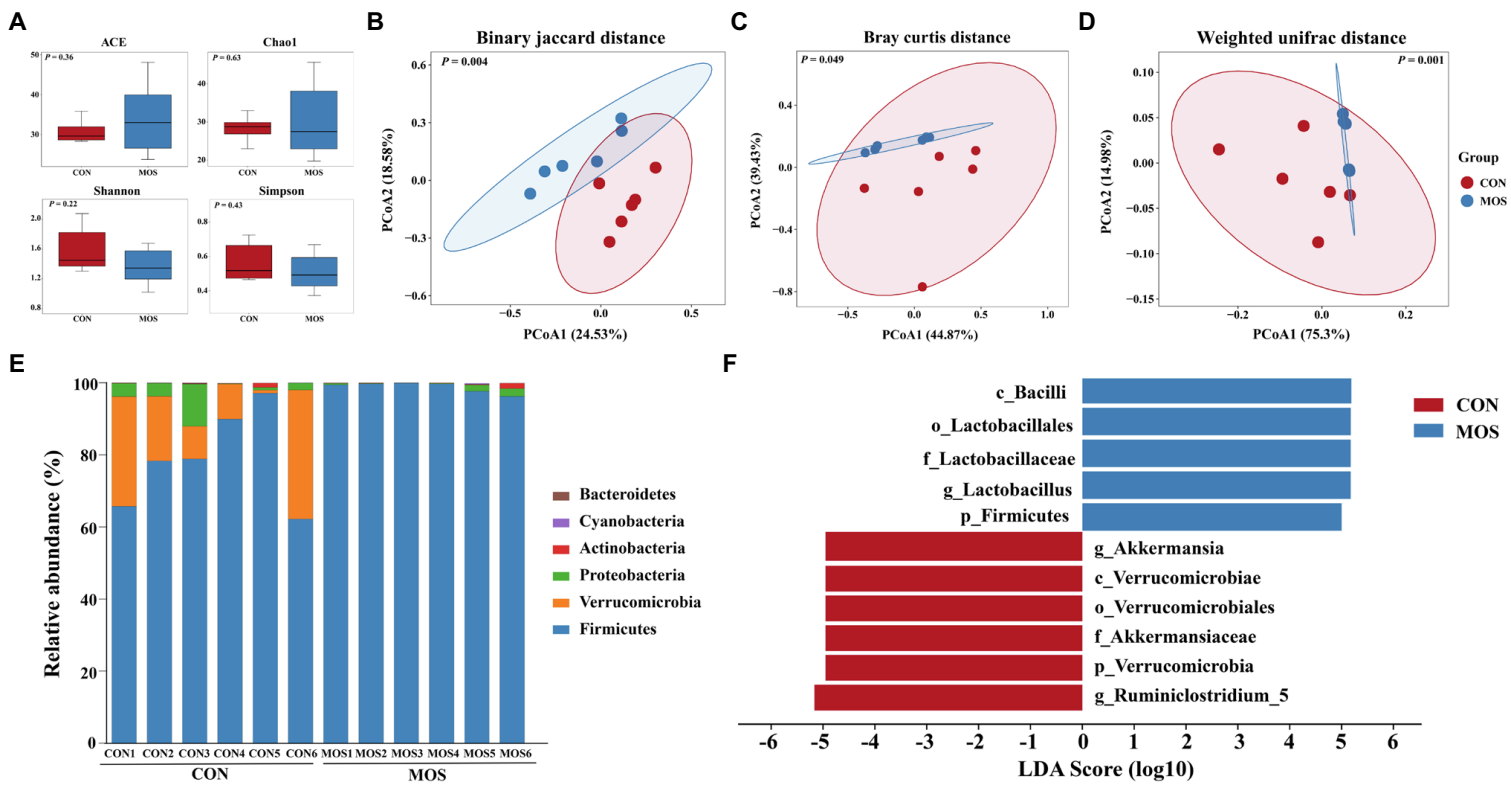
Alpha diversity, beta diversity, and taxonomic analysis of the microbial communities in the ileal digesta samples of neonatal goats in the CON and MOS groups. **(A)** Alpha diversity index; Principal coordinate analysis (PCoA) score plots of the ileal microbiota based on the **(B)** Binary jaccard distance, **(C)** Baray curtis distance, and **(D)** weighted UniFrac distance; **(E)** The relative abundance of the microbial composition at the phylum level; **(F)** Linear discriminant analysis (LDA) effect size linear discriminant analysis (LEfSe) of the ileal microbiota. *p < 0.05, **p < 0.01.

Supplementation with MOS exerted antioxidant and anti-inflammatory effect on young goats, we further investigated whether MOS could change the ileal microbiota that may influence goat’s health in early life. A total of 557,276 bacterial 16S rRNA sequence raw reads were obtained from 12 ileal digesta samples, with an average of 46,439.67 reads per sample. After quality control, 539,585 clean reads were retained, of which the total number of unique and clustered into representative bacterial OTU reads was 310,782, with an average of 25,898.50 reads per sample (Supplementary Table S2). The results of α-diversity showed that no differences (*p* > 0.10) in Chao1, Ace, Shannon, and Simpson index were found between the CON and MOS groups (Figure 4A). However, a distinct separation between the two group was observed in the PCoA plot based on Binary jaccard (*p* = 0.004), Bray curtis (*p* = 0.049) and weighted UniFrac distance (*p* = 0.001; Figures 4B–D). Taxonomic analysis revealed a total of six phyla were identified in both groups from 12 ileal digesta samples (Figure 4E). Among them, the *Firmicutes* was the dominant bacteria in the ileal digesta of neonatal goats, which accounted for more than 78 and 98% of total bacteria in the CON and MOS groups, respectively. Additionally, the *Verrucomicrobia* accounted for more than 17% in the CON group (Figure 4E). The relative abundance of *Firmicutes* was greater (*p* = 0.006) in the MOS group than that in the CON group; however, the relative abundance of *Verrucomicrobia* was significantly higher (*p* = 0.004) in the CON group than in the MOS group (Table 5). Compared with the CON group, the relative abundance of *Proteobacteria* tended to be lower (*p* = 0.078) in the MOS group (Table 5).

**Table 5:** The relative abundance of bacteria in the ileal digesta samples at phylum level.

Phylum	Treatment		SEM	*p*-value
	CON	MOS		
*Firmicutes*	78.690	98.783	4.016	0.006
*Verrucomicrobia*	17.309	0.002	3.705	0.004
*Proteobacteria*	3.638	0.769	0.939	0.078
*Actinobacteria*	0.272	0.331	0.144	0.631
*Bacteroidetes*	0.066	0.034	0.021	0.298
*Cyanobacteria*	0.025	0.081	0.023	0.471

*Values are expressed as means ± SEM, n = 6. p < 0.05 was regarded as statistically significant, and 0.05 < p < 0.10 was regarded as a statistical tendency*.

At the genus level, a total of 41 genera were detected in the ileal digesta samples of neonatal goats and 29 genera were identified in both groups, while 12 genera were only identified in the MOS group (Supplementary Table S3). The relative abundance of *Lactobacillus* was higher (*p* = 0.041) in the MOS group than that in the CON group; however, *Akkermansia* and *Ruminiclostridium_5* were significantly enriched (*p* = 0.004 and *p* = 0.025) in the CON group as compared with the MOS group (Table 6). Furthermore, the relative abundances of *Escherichia-Shigella* and *Lachnoclostridium* tended to be higher (*p* = 0.055 and *p* = 0.066) in the CON group than those in the MOS group (Table 6). The results of LEfSe analysis showed that MOS group characterized by a higher relative abundance of *Lactobacillus*, while CON group characterized by a higher relative abundance of *Akkermansia* and *Ruminiclostridium_5* (LDA > 2; Figure 4F).

**Table 6:** The relative abundance of bacteria in the ileal digesta samples at genus level (average relative abundance > 0.1% in at least one group).

Genus	Treatment		SEM	*p*-value
	CON	MOS		
*Lactobacillus*	66.871	97.100	8.016	0.010
*Akkermansia*	17.309	0.002	3.705	0.004
*Escherichia-Shigella*	3.539	0.326	0.948	0.055
*[Ruminococcus]_gnavus_group*	1.394	0.005	0.523	0.262
*Lachnoclostridium*	0.168	0.030	0.044	0.066
*Bifidobacterium*	0.247	0.043	0.089	0.128
*Ruminiclostridium_5*	0.100	0.002	0.029	0.025
*Streptococcus*	0.157	0.145	0.059	0.378
*Clostridium_sensu_stricto_1*	0.039	0.116	0.044	0.471
*Sarcina*	9.841	0.050	4.910	0.749
*Bacillus*	0	0.739	0.356	0.150
*uncultured_bacterium_f_Erysipelotrichaceae*	0	0.313	0.157	0.631
*Halomonas*	0	0.220	0.110	0.631
*Actinomyces*	0	0.145	0.072	0.631
*Trueperella*	0.025	0.143	0.051	0.262

*Values are expressed as means ± SEM, n = 6. p < 0.05 was regarded as statistically significant, and 0.05 < p < 0.10 was regarded as a statistical tendency*.

### Predicted the Metagenomic Metabolic Function of Microbiota in the Ileal Digesta

**Figure 5:**
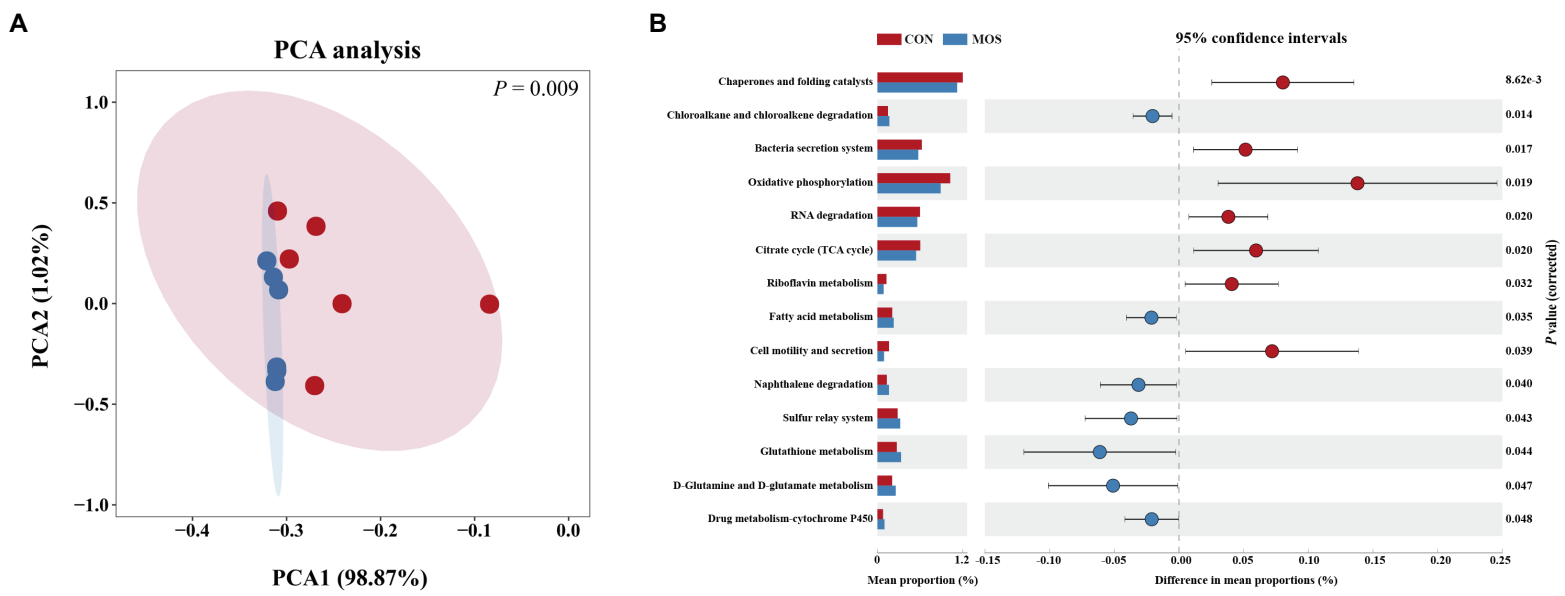
Metagenomic function predicted by PICRUSt2. **(A)** Principal component analysis (PCA) of functional pathways based on Kyoto Encyclopedia of Genes and Genomes (KEGG) level 3. **(B)** Significantly different KEGG pathways (level 3) detected between CON and MOS group.

To better understanding the molecular functional changes of ileal microbiota induced by MOS supplementation, we employed PICRUSt2 to predict the metagenomic contribution of identified microbial communities from the KEGG pathways. The PCA plot based on the relative abundance of KEGG level3 pathways demonstrated a distinct separation (*p* = 0.009) between CON and MOS groups (Figure 5A). A total of 250 functional pathways on KEGG level3 were detected and 14 pathways presented the significant difference between CON and MOS groups which accounted for 5.6% of the overall detected pathways. In details, the CON group had higher abundances of gene families involved in chaperones and folding catalysts, bacteria secretion system, oxidative phosphorylation, RNA degradation, TCA cycle, riboflavin metabolism, and cell motility and secretion, while the gene families of the MOS group were mainly enriched in chloroalkane and chloroalkene degradation, fatty acid metabolism, naphthalene degradation, sulfur relay system, glutathione metabolism, D-Glutamine and D-glutamate metabolism, and drug metabolism-cytochrome P450 (Figure 5B).

### Spearman’s Correlation Analysis Between Main Bacteria and Serum Antioxidant Indices, Inflammatory Cytokines as Well as sIgA in the Ileal Digesta

**Figure 6:**
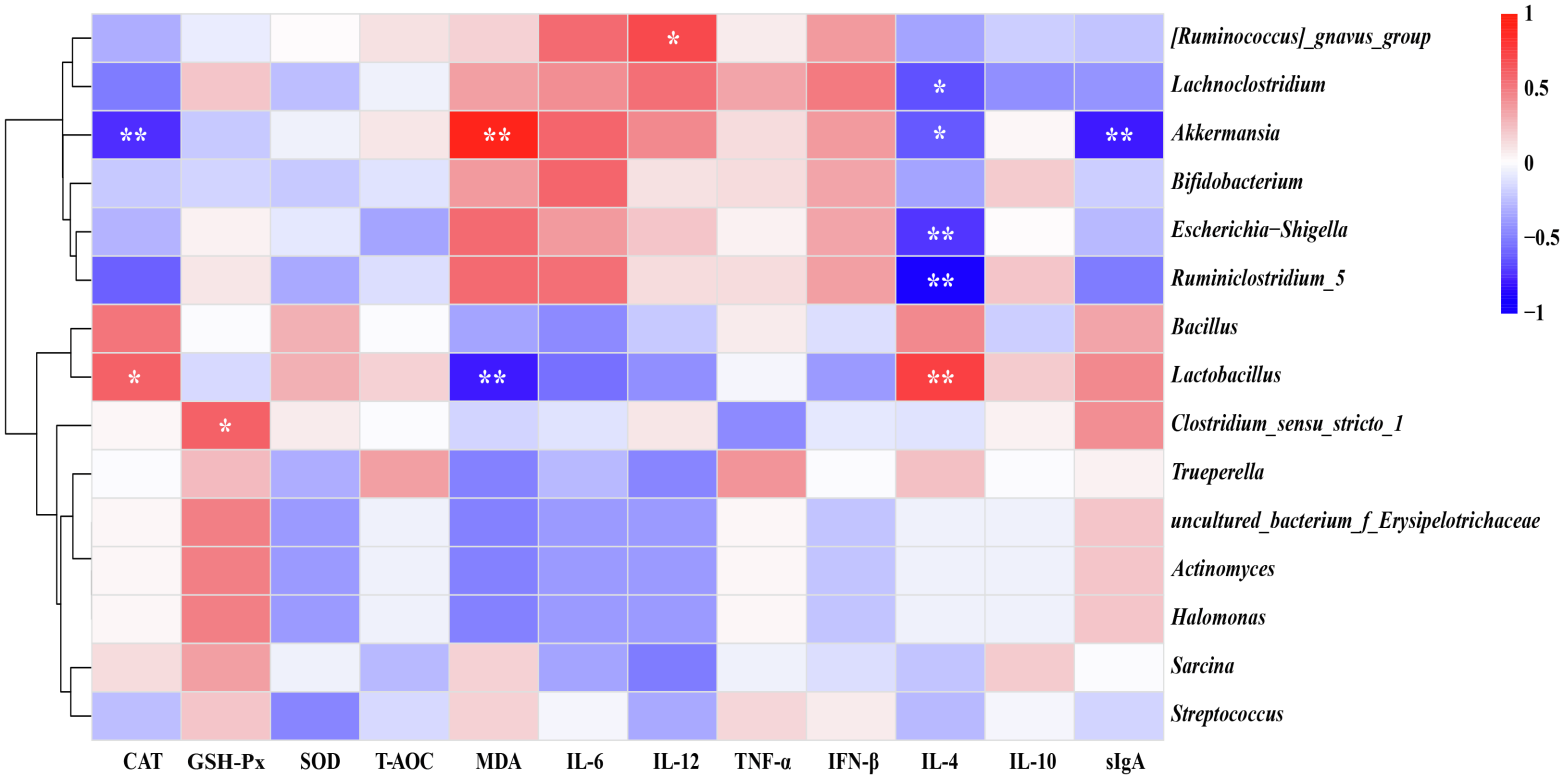
Heatmap of spearman’s correlation between ileal microbiota and serum antioxidative indices, inflammatory cytokines as well as secretory IgA (sIgA) level of the ileal digesta. The correlation was analyzed based on main bacteria (average relative abundance more than 0.1% in at least one group) at genus level. The blue suggests a negative correlation, and the red suggests a positive correlation. ^*^p < 0.05, ^**^p < 0.01.

As illustrated in Figure 6, Spearman’s correlation coefficient was performed to investigate relationship between the phenotypic values (serum antioxidant indices, inflammatory cytokines, and sIgA in the ileal digesta) and main bacteria (average relative abundance more than 0.1% in at least one group at the genus level). In details, *Lactobacillus* showed positive (*p* < 0.05) correlation with CAT and IL-4, while it was negatively correlated (*p* < 0.05) with MDA. *Akkermansia* was negatively correlated (*p* < 0.05) with CAT, IL-4, and sIgA but positively correlated (*p* < 0.05) with MDA. Furthermore, *Lachnoclostridium*, *Escherichia-Shigella*, and *Ruminiclostridium_5* were negatively correlated (*p* < 0.05) with IL-4. There had a significantly positive correlation (*p* < 0.05) between *Clostridium_sensu_stricto_1* and GSH-Px. Also, we found a remarkably positive correlation (*p* < 0.05) between *[Ruminococcus]_gnavus_group* and IL-12.

## Discussion

Functional oligosaccharides characterized by promoting immunity, suppressing pathogenic bacteria, and maintaining intestinal health have received extensive attention (Guerra-Ordaz et al., 2013; Tran et al., 2018). As a typical kind of functional oligosaccharides derived from the cell wall of yeast, MOS is widely used in animal feed to promote nutrient digestibility and alleviate intestinal disease (Spring et al., 2015; Duan et al., 2016). This study was the first time to clarify the effects of MOS supplementation on passive immunity transfer, intestinal development, and intestinal bacteria colonization in neonatal goats.

Regarding the dose of MOS, there were significant variations in different studies, and it ranged from 1 to 10 g per day (Swanson et al., 2002; Berge, 2016). In the current study, the amount of MOS supplementation was 0.06% of BW in the colostrum or milk replacer (an average inclusion of 1.41 g per day, purity: 99%), which referenced from two previous studies of calves (Heinrichs et al., 2013; Tóth et al., 2020). MOS supplementation did not affect the values of blood biochemical index value in the present trial, which was in agreement with the study of calves that the blood levels of TP, TG, CHOL, BUN, and GLU had no changes when supplemented 4 g MOS per day (Silva et al., 2012). Furthermore, the serum hormone levels of GH, INS, and IGF-1 were also unaffected by MOS supplementation. The above results may be attributed to the short experimental period and the growth stage of goats that underwent rapid growth and development during the first 1 week; thus, the influence induced by age highly concealed the contribution of MOS. GLP-1 plays a vital role in reducing gastric emptying and promoting the growth and development of small intestine. A higher GLP-1 level usually represents efficient digestion and absorption of dietary nutrients (Lim et al., 2009). The present study results showed that the serum level of GLP-1 decreased with MOS supplementation; however, little information about the effect of MOS supplementation on serum GLP-1 level was reported, and the mechanism deserved further study.

Newborn dairy calves received MOS in milk replacer at 0.6 g/kg BW presented a significant increase in serum IgG concentrations during the first 21 days of life compared with the control group (Lazarevic et al., 2010). Contradict with the above results, Brady et al. (2015) have shown that supplementing 30 g of MOS per day to newborn Holstein heifer and bull calves reduced serum IgG concentration at 24 h. Furthermore, a recent study in North American using 240 newborn Holstein dairy calves has reported that the addition of MOS to colostrum replacer did not affect serum IgG level at 24 h (Robichaud et al., 2014). Compared to the aforementioned studies, the current study set more sample collection points, and the results showed that only the serum IgG levels at 3 and 6 h were affected by MOS supplementation. Our results indicated that the reduction of serum IgG level of goats in the MOS group might be attributed to the less bacteria colonialization during the first 6 h and MOS adhering to intestinal epithelium delayed the passive transfer of IgG. Once beneficial bacteria rapidly colonialized, MOS could be utilized and the decrease of MOS adhered to epithelium eliminated the negative influence on IgG absorption (Flickinger et al., 2000).

As we all known, once the concentrations of reactive oxygen species surpassing the capacity of antioxidant, DNA, proteins, and endogenous lipids of cells will be widely damaged and finally induces inflammatory response (Yu, 1994). MDA is one of the essential indices to assess oxidative stress which damages the membrane structure and function of cells (Gawe et al., 2004). In contrast, GSH-Px and SOD as main enzymatic scavengers can counteract the adverse effects induced by oxidative stress, which are always recognized as the vital indicator of antioxidative capacity (Tabrez and Ahmad, 2009). Meanwhile, CAT can oxidize alcohol, formic acid, and phenols using H_2_O_2_ and it can also directly promote the conversion of H_2_O_2_ to H_2_O and O_2_ to alleviate the oxidative stress induced by the poisonous H_2_O_2_ accumulation (Xue et al., 2020). Previous studies have been proven that MOS exhibits good antioxidative properties, including increasing the serum levels of GSH-Px, SOD, and T-AOC and reducing serum MDA levels in sheep and chickens (Liu et al., 2015; Zheng et al., 2018; Zhou et al., 2019). Consistent with the above results, in the current study, MOS supplementation increased serum CAT level and reduced MDA level of neonatal goats. Meanwhile, Duan et al. (2016) demonstrated that MOS addition decreased serum concentrations of pro-inflammatory cytokines IL-2 and IL-4 but increased anti-inflammatory cytokine IL-10 level. Our results showed that MOS supplementation decreased the serum level of IL-6 and increased IL-4 concentration, suggesting that MOS could promote systemic immunity and suppress the inflammatory response in neonatal goats.

Intestinal barrier is the first line of body defensive system and the dysfunction of that barrier may destroy the integrity of the epithelium and finally increase the risk of pathogenic invasion (Camilleri et al., 2012). Meanwhile, the intestinal epithelium integrity is mainly modulated by the functional genes or proteins including Occludin, ZO-1, and Claudin. In this study, the relative expression of genes involved in the tight junction (*ZO-1*, *Claudin1*, and *Claudin2*) and inflammation (*TNF-α*, *IL-6*, and *IL-10*) was partly consistent with the findings of Che et al. (2011), which suggested that MOS supplementation could maintain the intestinal integrity and prevent from the pathogenic invasion of neonatal goats. The intestinal microbiota and host exist a cross-talk that intestinal microbiota regulates the host mucosal immunity, and the host can also manipulate the intestinal microbiota community (Zhang et al., 2017). sIgA acts as a bridge between intestinal microbiota and host mucosal immunity to defend the pathogenic bacteria colonization and invasion (Tlaskalová-Hogenová et al., 2004). In the current study, MOS addition promoted the sIgA concentration of ileal digesta, suggesting that MOS addition had the potential to enhance mucosal immunity and ultimately alleviate inflammation in the ileum of neonatal goats.

The intestinal epithelium possesses abundant mannose-specific receptors (MR) and FimH lectin of type I fimbriae in pathogens including *E. coli* and *Salmonella*, which can bind to MR, then cause the attachment of the above bacteria to the epithelium. Consequently, many pathogenic bacteria colonized in the epithelium to generate toxins that damage the balance between intestinal bacteria and induce the occurrence of disease (De Los Santos et al., 2007). Fortunately, MOS can prevent the adhesion of pathogenic bacteria to the mucosa by decreasing the number of available binding sites for fimbriae and finally ensuring the health of intestine (Ofek et al., 1977). In the current study, the relative abundance of *E. coli* tended to decrease in the MOS group, which indicated MOS supplementation restrained the adhesion and colonization of pathogenic bacteria in the mucosal epithelium. Otherwise, beneficial bacteria generally compete with pathogenic bacteria and MOS supplementation could increase the amount of *Lactobacillus* and *Bifidobacterium* in the jejunum, ileum, and caecal digesta in pigs and broiler chicken (Baurhoo et al., 2007; Poeikhampha and Bunchasak, 2011). The relative abundance of *Lactobacillus* in the current study was significantly increased by the MOS supplementation that may cause the lower abundance of *E. coli*. *Firmicutes* plays a crucial role in structural carbohydrate metabolism (Brulc et al., 2009). In this study, the relative abundance of *Firmicutes* was higher in the MOS group than that in the CON group, which might be attributed to the higher prevalence of *Lactobacillus*.

The *Verrucomicrobia* phylum inhabits in the gut of humans and animals has a close phylogenetical relationship to *Planctomycetes* and *Chlamydiae* and is primarily comprised of *Akkermansia* species (Wagner and Horn, 2006). A Gram-negative bacteria *Akkermansia muciniphila* isolated from human feces can degrade mucin of intestinal epithelium (Wagner and Horn, 2006), and the pili-like structural protein of the above bacteria can directly participate in the regulation of intestinal immunity and enhance the resistance of transportation across the intestinal epithelium (Ottman et al., 2017). In the current study, we found that MOS supplementation decreased the relative abundance of *Akkermansia*, but the mechanism remains unknown and that needs further study. A previous study showed that MOS treatment decreased the abundance of genera *Lachnoclostridium* and *Ruminiclostridium 5* in the caecum of broilers (Mesa et al., 2017), which is consistent with the present results. The above information indicated that MOS supplementation during the neonatal stage could promote the colonization of beneficial bacteria and inhibit the adhesion of pathogens to the intestinal mucosa. The predicted function of 16S rRNA gene profiles showed that the most abundant categories were the functions of amino acids metabolism in the MOS group, which is partly consistent with the previous study that carbohydrate, protein, and amino acid metabolism are necessary for microbial survival (Erickson et al., 2012). These indicated that MOS might promote dietary amino acid utilization to maintain intestinal health.

The results of Spearman’s correlation analysis presented that the relative abundance of *Lactobacillus* was positively correlated with serum IL-4 and CAT levels, while negatively correlated with MDA of serum. *Lactobacillus* degrades lactose and oligosaccharides of milk into lactate and other short-chain fatty acids. Those metabolites may involve in regulating mucosal immunity and decreasing oxidative stress (Hamer et al., 2008; Walter, 2008). *Lactobacillus reuteri*, one of the most abundant *Lactobacillus* species, has been proven to inhibit the secretion of pro-inflammatory cytokines and promote anti-inflammatory capacity (Christensen et al., 2002; Hu et al., 2021). These results imply that *Lactobacillus* can improve antioxidant capacity and alleviate the inflammation of neonatal goats. Intestinal mucus layer serves as a physical barrier to prevent the invasion and adhesion of pathogens, and only some commensal bacteria can colonize and obtain nutrients from that layer (Sicard et al., 2017). However, as mentioned above, *Akkermansia* can degrade mucin into small molecule metabolites, which may increase the risk of pathogenic invasion. The above information may explain why *Akkermansia* was negatively correlated with CAT, IL4, and sIgA levels, but was positively correlated with MDA levels. Otherwise, lipopolysaccharide derived from the cell wall of gram-negative bacteria (*E. coli* and *Salmonella*) induces the inflammatory response of animal through activating the NF-κB signaling pathway to release pro-inflammatory cytokines (IL-6, IL-12, and TNF-α; Burgueño and Abreu, 2020). The negative correlation between the *E. coil* and IL-4 may be due to the serum IL-4 level suppressed by release the above proinflammatory cytokines induced by the *E. coil*. The genus *Clostridium_sensu_stricto_1* belonging to *Clostridia* bacteria, can use dietary carbohydrate to produce butyrate (Pei et al., 2021). It has been reported that butyrate exerts positive effects on the reduction of oxidative stress (Hamer et al., 2008), which is in line with the current study that showed a positive correlation between *Clostridium_sensu_stricto_1* and serum GSH-Px level.

## Conclusion

Feeding MOS to the neonatal goats in the first 6 h after colostrum feeding reduced passive transfer of IgG, promoted serum antioxidative capacity and decreased serum levels of inflammatory cytokines. Furthermore, MOS supplementation improved intestinal integrity and mucosal immunity of the ileum by inducing sIgA secretion, modulation of the anti-inflammatory and pro-inflammatory gene expressions. MOS supplementation contributed to model a beneficial bacteria composition, which was reflected by the increased relative of abundance of *Lactobacillus* and decreased abundance of *Akkermansia*, *Ruminiclostridium_5*, *Escherichia-Shigella*, and *Lachnoclostridium*. Thus, MOS induced positive effects were more pronounced in neonatal goats that might be an effective approach to maintain intestinal health and improved the surviving rate of neonatal ruminants.

## Data Availability Statement

The datasets presented in this study can be found in online repositories. The names of the repository/repositories and accession number(s) can be found in the article/**Supplementary Material**.

## Ethics Statement

The animal study was reviewed and approved by Animal Ethics Committee of Institute of Subtropical Agriculture, Chinese Academy of Sciences. Written informed consent was obtained from the owners for the participation of their animals in this study.

## Author Contributions

CY, ZH, and ZT contributed to conception and design of the study. CY, TZ, QT, GL, YC, and KG collected the samples. CY, TZ, GL, and YC conducted laboratory analyses. CY performed the statistical analysis and wrote the manuscript. KG, ZT, and ZH revised the manuscript. All authors contributed to the article and approved the submitted version.

## Funding

This study was supported by National Natural Science Foundation of China (31772631 and 32072760), Hunan Key Research and Development Program (2020NK2049), and Innovation Province Project (2019RS3021).

## Conflict of Interest

The authors declare that the research was conducted in the absence of any commercial or financial relationships that could be construed as a potential conflict of interest.

## Publisher’s Note

All claims expressed in this article are solely those of the authors and do not necessarily represent those of their affiliated organizations, or those of the publisher, the editors and the reviewers. Any product that may be evaluated in this article, or claim that may be made by its manufacturer, is not guaranteed or endorsed by the publisher.

## Footnotes

^1^ https://www.cusabio.com/

^2^ http://www.bjapn.com

^3^ http://drive5.com/uparse

## References

[ref1] Abdel-Hamid T. M. Farahat M. H. (2016). Effect of dietary mannan-oligosaccharides on some blood biochemical, haematological parameters and carcass traits in purebred New Zealand white and crossbred rabbits. Anim. Prod. Sci. 56:2133. doi: 10.1071/AN15032

[ref2] Azizzadeh M. Shooroki H. F. Kamalabadi A. S. Stevenson M. A. (2012). Factors affecting calf mortality in Iranian Holstein dairy herds. Prev. Vet. Med. 104, 335–340. doi: 10.1016/j.prevetmed.2011.12.007, PMID: 22230657

[ref3] Baurhoo B. Phillip L. Ruiz-Feria C. (2007). Effects of purified lignin and mannan oligosaccharides on intestinal integrity and microbial populations in the ceca and litter of broiler chickens. Poult. Sci. 86, 1070–1078. doi: 10.1093/ps/86.6.1070, PMID: 17495075

[ref4] Berge A. (2016). A meta-analysis of the inclusion of bio-Mos® in milk or milk replacer fed to dairy calves on daily weight gain in the pre-weaning period. J. Anim. Res. Nutr. 01, 1–7. doi: 10.21767/2572-5459.100020

[ref5] Blacklaws B. A. Berriatua E. Torsteinsdottir S. Watt N. J. Andres D. D. Klein D.. (2004). Transmission of small ruminant lentiviruses. Vet. Microbiol. 101, 199–208. doi: 10.1016/j.vetmic.2004.04.006, PMID: 15223124

[ref6] Borghesi J. Mario L. C. Rodrigues M. N. Favaron P. O. Miglino M. A. (2014). Immunoglobulin transport during gestation in domestic animals and humans—a review. Open J. Anim. Sci. 04, 323–336. doi: 10.4236/ojas.2014.45041

[ref7] Bozkurt M. Bintaş E. Kırkan Ş. Akşit H. Küçükyılmaz K. Erbaş G.. (2016). Comparative evaluation of dietary supplementation with mannan oligosaccharide and oregano essential oil in forced molted and fully fed laying hens between 82 and 106 weeks of age. Poult. Sci. 95, 2576–2591. doi: 10.3382/ps/pew140, PMID: 27143766

[ref8] Brady M. Godden S. Haines D. (2015). Supplementing fresh bovine colostrum with gut-active carbohydrates reduces passive transfer of immunoglobulin G in Holstein dairy calves. J. Dairy Sci. 98, 6415–6422. doi: 10.3168/jds.2015-9481, PMID: 26117349

[ref9] Brulc J. M. Antonopoulos D. A. Miller M. E. B. Wilson M. K. Yannarell A. C. Dinsdale E. A.. (2009). Gene-centric metagenomics of the fiber-adherent bovine rumen microbiome reveals forage specific glycoside hydrolases. Proc. Natl. Acad. Sci. U. S. A. 106, 1948–1953. doi: 10.1073/pnas.0806191105, PMID: 19181843 PMC2633212

[ref10] Burgueño J. F. Abreu M. T. (2020). Epithelial toll-like receptors and their role in gut homeostasis and disease. Nat. Rev. Gastroenterol. Hepatol. 17, 263–278. doi: 10.1038/s41575-019-0261-4, PMID: 32103203

[ref11] Camilleri M. Madsen K. Spiller R. Meerveld B. Verne G. N. (2012). Intestinal barrier function in health and gastrointestinal disease. Neurogastroenterol. Motil. 24, 503–512. doi: 10.1111/j.1365-2982.2012.01921.x, PMID: 22583600 PMC5595063

[ref12] Caporaso J. G. Kuczynski J. Stombaugh J. Bittinger K. Bushman F. D. Costello E. K.. (2010). QIIME allows analysis of high-throughput community sequencing data. Nat. Methods 7, 335–336. doi: 10.1038/nmeth.f.303, PMID: 20383131 PMC3156573

[ref13] Che T. Johnson R. Kelley K. Van Alstine W. Dawson K. Moran C.. (2011). Mannan oligosaccharide improves immune responses and growth efficiency of nursery pigs experimentally infected with porcine reproductive and respiratory syndrome virus. J. Anim. Sci. 89, 2592–2602. doi: 10.2527/jas.2010-3208, PMID: 21454863

[ref14] Cheng Y. Yang C. Tan Z. L. He Z. X. (2021). Changes of intestinal oxidative stress, inflammation, and gene expression in neonatal Diarrhoea kids. Front. Vet. Sci. 8:598691. doi: 10.3389/fvets.2021.598691, PMID: 33614759 PMC7890263

[ref15] Christensen H. R. Frokiaer H. Pestka J. J. (2002). Lactobacilli differentially modulate expression of cytokines and maturation surface markers in murine dendritic cells. J. Immunol. 168, 171–178. doi: 10.4049/jimmunol.168.1.171, PMID: 11751960

[ref16] De Los Santos F. S. Donoghue A. Farnell M. Huff G. Huff W. Donoghue D. (2007). Gastrointestinal maturation is accelerated in Turkey poults supplemented with a mannan-oligosaccharide yeast extract (Alphamune). Poult. Sci. 86, 921–930. doi: 10.1093/ps/86.5.921, PMID: 17435027

[ref17] Donahue M. Godden S. M. Bey R. Wells S. Oakes J. M. Sreevatsan S.. (2012). Heat treatment of colostrum on commercial dairy farms decreases colostrum microbial counts while maintaining colostrum immunoglobulin G concentrations. J. Dairy Sci. 95, 2697–2702. doi: 10.3168/jds.2011-5220, PMID: 22541498

[ref18] Duan X. D. Chen D. W. Zheng P. Tian G. Wang J. P. Mao X. B.. (2016). Effects of dietary mannan oligosaccharide supplementation on performance and immune response of sows and their offspring. Anim. Feed Sci. Technol. 218, 17–25. doi: 10.1016/j.anifeedsci.2016.05.002

[ref19] Dwyer C. M. Conington J. Corbiere F. Holmoy I. H. Muri K. Nowak R.. (2016). Invited review: improving neonatal survival in small ruminants: science into practice. Animal 10, 449–459. doi: 10.1017/s1751731115001974, PMID: 26434788

[ref20] Erickson A. R. Cantarel B. L. Lamendella R. Darzi Y. Mongodin E. F. Pan C.. (2012). Integrated metagenomics/metaproteomics reveals human host-microbiota signatures of Crohn's disease. PLoS One 7:e49138. doi: 10.1371/journal.pone.0049138, PMID: 23209564 PMC3509130

[ref21] Fischer A. J. Song Y. He Z. Haines D. M. Guan L. L. Steele M. A. (2018). Effect of delaying colostrum feeding on passive transfer and intestinal bacterial colonization in neonatal male Holstein calves. J. Dairy Sci. 101, 3099–3109. doi: 10.3168/jds.2017-13397, PMID: 29397179

[ref22] Flickinger E. A. Wolf B. W. Garleb K. A. Chow J. Leyer G. J. Johns P. W.. (2000). Glucose-based oligosaccharides exhibit different in vitro fermentation patterns and affect in vivo apparent nutrient digestibility and microbial populations in dogs. J. Nutr. 130, 1267–1273. doi: 10.1038/sj.ijo.0801233, PMID: 10801928

[ref23] Franklin S. T. Newman M. C. Newman K. E. Meek K. I. (2005). Immune parameters of dry cows fed Mannan oligosaccharide and subsequent transfer of immunity to calves. J. Dairy Sci. 88, 766–775. doi: 10.3168/jds.S0022-0302(05)72740-5, PMID: 15653543 PMC7190086

[ref24] Gawe S. Wardas M. Niedworok E. Wardas P. (2004). Malondialdehyde (MDA) as a lipid peroxidation marker. Wiad. Lek. 57, 453–455. PMID: 15765761

[ref25] Godden S. M. Smolenski D. J. Donahue M. Oakes J. M. Bey R. Wells S.. (2012). Heat-treated colostrum and reduced morbidity in preweaned dairy calves: results of a randomized trial and examination of mechanisms of effectiveness. J. Dairy Sci. 95, 4029–4040. doi: 10.3168/jds.2011-5275, PMID: 22720957

[ref26] Guerra-Ordaz A. Molist F. Hermes R. de Segura A. G. La Ragione R. Woodward M.. (2013). Effect of inclusion of lactulose and *Lactobacillus plantarum* on the intestinal environment and performance of piglets at weaning. Anim. Feed Sci. Technol. 185, 160–168. doi: 10.1016/j.anifeedsci.2013.07.009

[ref27] Hamer H. M. Jonkers D. Venema K. Vanhoutvin S. Troost F. J. Brummer R.-J. (2008). Review article: the role of butyrate on colonic function. Aliment. Pharmacol. Ther. 27, 104–119. doi: 10.1111/j.1365-2036.2007.03562.x, PMID: 17973645

[ref28] Heinrichs A. Heinrichs B. Jones C. (2013). Fecal and saliva IgA secretion when feeding a concentrated mannan oligosaccharide to neonatal dairy calves. Prof. Anim. Sci. 29, 457–462. doi: 10.15232/S1080-7446(15)30266-7

[ref29] Hu R. Lin H. Wang M. Zhao Y. Yang M. (2021). *Lactobacillus reuteri*-derived extracellular vesicles maintain intestinal immune homeostasis against lipopolysaccharide-induced inflammatory responses in broilers. J. Anim. Sci. Biotechnol. 12:25. doi: 10.1186/s40104-020-00532-4, PMID: 33593426 PMC7888134

[ref30] Hunter P. R. Thompson R. (2005). The zoonotic transmission of giardia and cryptosporidium. Int. J. Parasitol. 35, 1181–1190. doi: 10.1016/j.ijpara.2005.07.009, PMID: 16159658

[ref31] Langille M. Zaneveld J. Caporaso J. G. Mcdonald D. Knights D. Reyes J. A.. (2013). Predictive functional profiling of microbial communities using 16S rRNA marker gene sequences. Nat. Biotechnol. 31, 814–821. doi: 10.1038/nbt.2676, PMID: 23975157 PMC3819121

[ref32] Lazarevic M. Spring P. Shabanovic M. Tokic V. Tucker L. (2010). Effect of gut active carbohydrates on plasma IgG concentrations in piglets and calves. Animal 4, 938–943. doi: 10.1017/s1751731110000194, PMID: 22444266

[ref33] Lim G. E. Huang G. J. Flora N. LeRoith D. Rhodes C. J. Brubaker P. L. (2009). Insulin regulates glucagon-like peptide-1 secretion from the enteroendocrine L cell. Endocrinology 150, 580–591. doi: 10.1210/en.2008-0726, PMID: 18818290 PMC5393261

[ref34] Liu J. Xu Q. Zhang J. Zhou X. Lyu F. Zhao P.. (2015). Preparation, composition analysis and antioxidant activities of konjac oligo-glucomannan. Carbohydr. Polym. 130, 398–404. doi: 10.1016/j.carbpol.2015.05.025, PMID: 26076641

[ref35] Lucey P. M. Lean I. J. Aly S. S. Golder H. M. Rossow H. A. (2021). Effects of mannan-oligosaccharide and *Bacillus subtilis* supplementation to preweaning Holstein dairy heifers on body weight gain, diarrhea, and shedding of fecal pathogens. J. Dairy Sci. 104, 4290–4302. doi: 10.3168/jds.2020-19425, PMID: 33752289

[ref36] Malmuthuge N. Chen Y. Liang G. Goonewardene L. A. Guan L. L. (2015). Heat-treated colostrum feeding promotes beneficial bacteria colonization in the small intestine of neonatal calves. J. Dairy Sci. 98, 8044–8053. doi: 10.3168/jds.2015-9607, PMID: 26342981

[ref37] Mesa D. Lammel D. R. Balsanelli E. Sena C. Noseda M. D. Caron L. F.. (2017). Cecal microbiota in broilers fed with prebiotics. Front. Genet. 8:153. doi: 10.3389/fgene.2017.00153, PMID: 29089963 PMC5650999

[ref38] Micha S. Patrycja S. Jakub F. (2020). Supplementation of bovine colostrum in inflammatory bowel disease: benefits and contraindications. Adv. Nutr. 12, 533–545. doi: 10.1093/advances/nmaa120, PMID: 33070186 PMC8009748

[ref39] Morrill K. M. Conrad E. Lago A. Campbell J. Quigley J. Tyler H. (2012). Nationwide evaluation of quality and composition of colostrum on dairy farms in the United States. J. Dairy Sci. 95, 3997–4005. doi: 10.3168/jds.2011-5174, PMID: 22720954

[ref40] Nordi W. M. Moretti D. B. Lima A. L. Pauletti P. Susin I. Machado-Neto R. (2012). Intestinal IgG uptake by small intestine of goat kid fed goat or lyophilized bovine colostrum. Livest. Sci. 144, 205–210. doi: 10.1016/j.livsci.2011.11.017

[ref41] Ofek I. Mirelman D. Sharon N. (1977). Adherence of *Escherichia coli* to human mucosal cells mediated by mannose receptors. Nature 265, 623–625. doi: 10.1038/265623a0, PMID: 323718

[ref42] Ottman N. Reunanen J. Meijerink M. Pietilä T. E. Kainulainen V. Klievink J.. (2017). Pili-like proteins of *Akkermansia muciniphila* modulate host immune responses and gut barrier function. PLoS One 12:e0173004. doi: 10.1371/journal.pone.0173004, PMID: 28249045 PMC5332112

[ref43] Parks D. H. Tyson G. W. Philip H. Beiko R. G. (2014). STAMP: statistical analysis of taxonomic and functional profiles. Bioinformatics 30, 3123–3124. doi: 10.1093/bioinformatics/btu494, PMID: 25061070 PMC4609014

[ref44] Pei Y. Chen C. Mu Y. Yang Y. Li K. (2021). Integrated microbiome and metabolome analysis reveals a positive change in the intestinal environment of myostatin edited large white pigs. Front. Microbiol. 12:628685. doi: 10.3389/fmicb.2021.628685, PMID: 33679652 PMC7925633

[ref45] Poeikhampha T. Bunchasak C. (2011). Comparative effects of sodium gluconate, mannan oligosaccharide and potassium diformate on growth performances and small intestinal morphology of nursery pigs. Asian Australas. J. Anim. Sci. 24, 844–850. doi: 10.5713/ajas.2011.10334

[ref46] Quast C. Pruesse E. Yilmaz P. Gerken J. Glckner F. O. (2012). The SILVA ribosomal RNA gene database project: improved data processing and web-based tools. Nucleic Acids Res. 41, D590–D596. doi: 10.1093/nar/gks1219, PMID: 23193283 PMC3531112

[ref47] Robichaud M. V. Godden S. Haines D. Haley D. Pearl D. (2014). Addition of gut active carbohydrates to colostrum replacer does not improve passive transfer of immunoglobulin G in Holstein dairy calves. J. Dairy Sci. 97, 5700–5708. doi: 10.3168/jds.2013-7854, PMID: 25022688

[ref48] Schmittgen T. D. Livak K. J. (2008). Analyzing real-time PCR data by the comparative C(T) method. Nat. Protoc. 3, 1101–1108. doi: 10.1038/nprot.2008.73, PMID: 18546601

[ref49] Sicard J.-F. Le Bihan G. Vogeleer P. Jacques M. Harel J. (2017). Interactions of intestinal bacteria with components of the intestinal mucus. Front. Cell. Infect. Microbiol. 7:387. doi: 10.3389/fcimb.2017.00387, PMID: 28929087 PMC5591952

[ref50] Silva J. T. D. Bittar C. M. M. Ferreira L. S. (2012). Evaluation of mannan-oligosaccharides offered in milk replacers or calf starters and their effect on performance and rumen development of dairy calves. Rev. Bras. Zootec. 41, 746–752. doi: 10.1590/S1516-35982012000300038

[ref51] Singh M. K. Rai B. Sharma N. (2011). Factors affecting survivability of Jamunapari kids under semi-intensive management system. Indian J. Anim. Sci. 34, 204–216. doi: 10.1016/j.domaniend.2007.03.001

[ref52] Spring P. Wenk C. Connolly A. Kiers A. (2015). A review of 733 published trials on bio-Mos®, a mannan oligosaccharide, and Actigen®, a second generation mannose rich fraction, on farm and companion animals. J. Appl. Anim. Nutr. 3, 746–752. doi: 10.1017/jan.2015.6

[ref53] Swanson K. S. Grieshop C. M. Flickinger E. A. Bauer L. L. Healy H.-P. Dawson K. A.. (2002). Supplemental fructooligosaccharides and mannanoligosaccharides influence immune function, ileal and total tract nutrient digestibilities, microbial populations and concentrations of protein catabolites in the large bowel of dogs. J. Nutr. 132, 980–989. doi: 10.1046/j.1365-277X.2002.00398.x, PMID: 11983825

[ref54] Tabrez S. Ahmad M. (2009). Effect of wastewater intake on antioxidant and marker enzymes of tissue damage in rat tissues: implications for the use of biochemical markers. Food Chem. Toxicol. 47, 2465–2478. doi: 10.1016/j.fct.2009.07.004, PMID: 19596398

[ref55] Thiruvenkadan A. K. Karunanithi K. (2007). Mortality and replacement rate of Tellicherry and its crossbred goats in Tamil Nadu. Indian J. Anim. Sci. 39, 465–479. doi: 10.1051/gse:2007015

[ref56] Tlaskalová-Hogenová H. Štěpánková R. Hudcovic T. Tučková L. Cukrowska B. Lodinová-Žádnıková R.. (2004). Commensal bacteria (normal microflora), mucosal immunity and chronic inflammatory and autoimmune diseases. Immunol. Lett. 93, 97–108. doi: 10.1016/j.imlet.2004.02.005, PMID: 15158604

[ref57] Tóth S. Kovács M. Bóta B. Szabó-Fodor J. Bakos G. Fébel H. (2020). Effect of mannanoligosaccharide (MOS) and inulin supplementation on the performance and certain physiological parameters of calves reared on milk replacer. J. Appl. Anim. Res. 48, 228–234. doi: 10.1080/09712119.2020.1770096

[ref58] Tran T. H. T. Everaert N. Bindelle J. (2018). Review on the effects of potential prebiotics on controlling intestinal enteropathogens *Salmonella* and *Escherichia coli* in pig production. J. Anim. Physiol. Anim. Nutr. 102, 17–32. doi: 10.1111/jpn.12666, PMID: 28028851

[ref59] Wagner M. Horn M. (2006). The Planctomycetes, Verrucomicrobia, Chlamydiae and sister phyla comprise a superphylum with biotechnological and medical relevance. Curr. Opin. Biotechnol. 17, 241–249. doi: 10.1016/j.copbio.2006.05.005, PMID: 16704931

[ref60] Walter J. (2008). Ecological role of lactobacilli in the gastrointestinal tract: implications for fundamental and biomedical research. Appl. Environ. Microbiol. 74, 4985–4996. doi: 10.1128/AEM.00753-08, PMID: 18539818 PMC2519286

[ref61] Windeyer M. C. Leslie K. E. Godden S. M. Hodgins D. C. Lissemore K. D. Leblanc S. J. (2014). Factors associated with morbidity, mortality, and growth of dairy heifer calves up to 3 months of age. Prev. Vet. Med. 113, 231–240. doi: 10.1016/j.prevetmed.2013.10.019, PMID: 24269039

[ref62] Xue Y. Guo C. Hu F. Zhu W. Mao S. (2020). Undernutrition-induced lipid metabolism disorder triggers oxidative stress in maternal and fetal livers using a model of pregnant sheep. FASEB J. 34, 6508–6520. doi: 10.1096/fj.201902537R, PMID: 32232897

[ref63] Yang C. Cheng Y. Li X. Li H. Tan Z. (2021). Effects of dietary *Macleaya cordata* extract inclusion on transcriptomes and inflammatory response in the lower gut of early weaned goats. Anim. Feed Sci. Technol. 272:114792. doi: 10.1016/j.anifeedsci.2020.114792

[ref64] Yu B. P. (1994). Cellular defenses against damage from reactive oxygen species. Physiol. Rev. 74, 139–162. doi: 10.1152/physrev.1994.74.1.139, PMID: 8295932

[ref65] Zhang M. Sun K. Wu Y. Yang Y. Tso P. Wu Z. (2017). Interactions between intestinal microbiota and host immune response in inflammatory bowel disease. Front. Immunol. 8:942. doi: 10.3389/fimmu.2017.00942, PMID: 28855901 PMC5558048

[ref66] Zheng C. Li F. Hao Z. Liu T. (2018). Effects of adding mannan oligosaccharides on digestibility and metabolism of nutrients, ruminal fermentation parameters, immunity, and antioxidant capacity of sheep. J. Anim. Sci. 96, 284–292. doi: 10.1093/jas/skx040, PMID: 29385475 PMC6140840

[ref67] Zhou M. Tao Y. Lai C. Huang C. Zhou Y. Yong Q. (2019). Effects of mannanoligosaccharide supplementation on the growth performance, immunity, and oxidative status of partridge shank chickens. Animals 9:817. doi: 10.3390/ani9100817, PMID: 31623222 PMC6827142

