# Supplementary material for: Supplementing Mannan Oligosaccharide Reduces the Passive Transfer of Immunoglobulin G and Improves Antioxidative Capacity, Immunity, and Intestinal Microbiota in Neonatal Goats
Source: Front Microbiol. 2022 Jan 4;12:795081. doi: 10.3389/fmicb.2021.795081 (PMC8764366; doi:10.3389/fmicb.2021.795081)

**Supplementary Table S1** Primers information for the genes used in quantitative PCR.

| Gene Name | Sequence (5' to 3') | Product size | Accession number |
| --- | --- | --- | --- |
| *Occludin* | ACTACGCACCAAGCAATGAC | 198 bp | XM_005694596.2 |
|  | AAGAGTGGAGGCAACACAGG |  |  |
| *ZO-1* | CGACCAGATCCTCAGGGTAA | 161 bp | XM_018066114.1 |
|  | AATCACCCACATCGGATTCT |  |  |
| *Claudin1* | CAGCATGGTATGGCAATAGA | 183 bp | XM_005675123.2 |
|  | GCCTGGGTGTTGGGTAAG |  |  |
| *Claudin2* | TACCCAACCCTCAACTCCAG | 166 bp | XM_005700206.3 |
|  | AAGAGCCAACCTCAGCAAGA |  |  |
| *IFN-β* | GGAAGATGCCGTATTGGT | 157 bp | JX458085.1 |
|  | TTCCTTCTGGATTGGCTC |  |  |
| *TNF-α* | CCACTGACGGGCTTTACCT | 141 bp | AY304502.1 |
|  | TGATGGCAGAGAGGATGTTG |  |  |
| *IL-1β* | AAGGCTCTCCACCTCCTCTC | 114 bp | DQ837160.1 |
|  | TTGTCCCTGATACCCAAGG |  |  |
| *IL-4* | GCTGGTCTGCTTACTGG | 103 bp | NM_001285681.1 |
|  | CTGCGATGTGAGGATGT |  |  |
| *IL-6* | TGACTTCTGCTTTCCCTACCC | 193 bp | HM565937.1 |
|  | GCCAGTGTCTCCTTGCTGTT |  |  |
| *IL-10* | GCTGTTGCCTGGTCTTCCT | 178 bp | DQ837159.1 |
|  | TGTTCAGTTGGTCCTTCATTTG |  |  |
| *IL-12* | ATTGAGGTCGTGATGGAAGC | 190 bp | NM_001285700.1 |
|  | GGGAGAAGTAGGAATGTGGG |  |  |
| *GAPDH* | TTCCACGGCACAGTCAAG | 116 bp | AJ431207.1 |
|  | TACTCAGCACCAGCATCACC |  |  |
| *β-actin* | ACCACTGGCATTGTCATGGACTCT | 200 bp | AF481159.1 |
|  | TCCTTGATGTCACGGACGATTTCC |  |  |

**Supplementary Table S2** Overview of 16s rDNA sequence data.

| Sample ID | Raw reads | Clean reads | Reads cluster in OTU | OTU number |
| --- | --- | --- | --- | --- |
| CON1 | 33525 | 33139 | 18860 | 17 |
| CON2 | 49029 | 47218 | 32155 | 12 |
| CON3 | 50940 | 50300 | 30215 | 41 |
| CON4 | 58276 | 57578 | 34536 | 40 |
| CON5 | 59140 | 56858 | 26831 | 68 |
| CON6 | 53121 | 51759 | 27740 | 40 |
| MOS1 | 37525 | 35954 | 20841 | 18 |
| MOS2 | 36813 | 34025 | 19029 | 18 |
| MOS3 | 51347 | 50334 | 29656 | 12 |
| MOS4 | 39800 | 39770 | 22745 | 19 |
| MOS5 | 37611 | 36758 | 26798 | 40 |
| MOS6 | 50149 | 45892 | 21376 | 44 |
| Sum | 557276 | 539585 | 310782 |  |
| Average | 46439.67 | 44965.42 | 25898.50 |  |

**Supplementary Table S3** Average relative abundance of bacteria in the ileal digesta at genus level

| Genus | Treatment | | *P* value |
| --- | --- | --- | --- |
|  | CON | MOS |  |
| *Lactobacillus* | 66.871 | 97.100 | 0.010 |
| *Akkermansia* | 17.309 | 0.002 | 0.004 |
| *Sarcina* | 9.841 | 0.050 | 0.749 |
| *Escherichia-Shigella* | 3.539 | 0.326 | 0.055 |
| *[Ruminococcus]_gnavus_group* | 1.394 | 0.005 | 0.262 |
| *Bifidobacterium* | 0.247 | 0.043 | 0.128 |
| *Lachnoclostridium* | 0.168 | 0.030 | 0.066 |
| *Streptococcus* | 0.157 | 0.145 | 0.378 |
| *Ruminiclostridium_5* | 0.100 | 0.002 | 0.025 |
| *Bacteroides* | 0.066 | 0.034 | 0.298 |
| *Clostridium_sensu_stricto_1* | 0.039 | 0.116 | 0.471 |
| *uncultured_bacterium_o_Lactobacillales* | 0.039 | 0.005 | 0.298 |
| *Bosea* | 0.032 | 0.059 | 0.810 |
| *Klebsiella* | 0.027 | 0.005 | 0.337 |
| *Trueperella* | 0.025 | 0.143 | 0.262 |
| *Phaseolus_acutifolius_tepary_bean* | 0.025 | 0.081 | 0.471 |
| *Lactococcus* | 0.021 | 0.027 | 0.522 |
| *Enterococcus* | 0.020 | 0.018 | 0.093 |
| *Clostridium_sensu_stricto_2* | 0.018 | 0.050 | 0.337 |
| *Sphingomonas* | 0.014 | 0.013 | 0.128 |
| *Blautia* | 0.013 | 0.011 | 0.936 |
| *Ochrobactrum* | 0.007 | 0.020 | 0.631 |
| *Pygmaiobacter* | 0.007 | 0.020 | 0.631 |
| *Caulobacter* | 0.004 | 0.020 | 0.522 |
| *Stenotrophomonas* | 0.004 | 0.014 | 0.378 |
| *uncultured_bacterium_f_Burkholderiaceae* | 0.004 | 0.013 | 0.873 |
| *uncultured_bacterium_f_Mitochondria* | 0.004 | 0.011 | 0.873 |
| *uncultured_bacterium_f_Rhizobiaceae* | 0.004 | 0.002 | 0.936 |
| *Ruminococcaceae_UCG-002* | 0.002 | 0.025 | 0.522 |
| *Bacillus* | 0 | 0.739 | 0.150 |
| *uncultured_bacterium_f_Erysipelotrichaceae* | 0 | 0.313 | 0.631 |
| *Halomonas* | 0 | 0.220 | 0.631 |
| *Actinomyces* | 0 | 0.145 | 0.631 |
| *Eisenbergiella* | 0 | 0.036 | 0.631 |
| *Globicatella* | 0 | 0.036 | 0.150 |
| *Allobaculum* | 0 | 0.034 | 0.631 |
| *Pseudomonas* | 0 | 0.027 | 0.337 |
| *Lysinibacillus* | 0 | 0.021 | 0.631 |
| *Allorhizobium-Neorhizobium-Pararhizobium-Rhizobium* | 0 | 0.014 | 0.631 |
| *Bradyrhizobium* | 0 | 0.014 | 0.337 |
| *Acinetobacter* | 0 | 0.013 | 0.631 |

**Supplementary Figure S1** Ileal morphology of neonatal goats in the **(A)** CON and **(B)** MOS group.


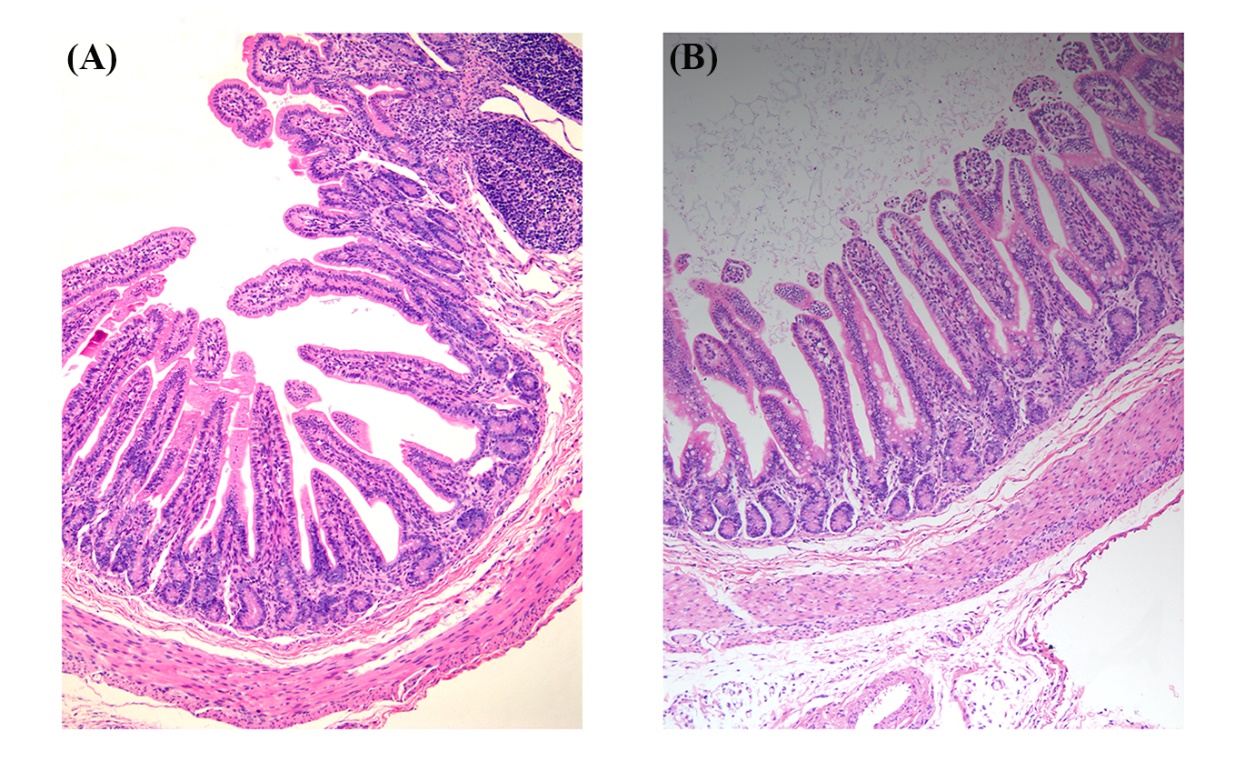

Supplement: Supplementary file 1 [file Data_Sheet_1.docx]
